# Case report: Understanding the impact of persistent tissue-localization of SARS-CoV-2 on immune response activity *via* spatial transcriptomic analysis of two cancer patients with COVID-19 co-morbidity

**DOI:** 10.3389/fimmu.2022.978760

**Published:** 2022-09-12

**Authors:** Mai Chan Lau, Yang Yi, Denise Goh, Chun Chau Lawrence Cheung, Benedict Tan, Jeffrey Chun Tatt Lim, Craig Ryan Joseph, Felicia Wee, Justina Nadia Lee, Xinru Lim, Chun Jye Lim, Wei Qiang Leow, Jing Yi Lee, Cedric Chuan Young Ng, Hamed Bashiri, Peng Chung Cheow, Chun Yip Chan, Ye Xin Koh, Thuan Tong Tan, Shirin Kalimuddin, Wai Meng David Tai, Jia Lin Ng, Jenny Guek-Hong Low, Tony Kiat Hon Lim, Jin Liu, Joe Poh Sheng Yeong

**Affiliations:** ^1^ Institute of Molecular and Cell Biology (IMCB), Agency of Science, Technology and Research (A*STAR), Singapore, Singapore; ^2^ Centre for Quantitative Medicine, Duke-NUS Medical School, Singapore, Singapore; ^3^ Department of Anatomical Pathology, Singapore General Hospital, Singapore, Singapore; ^4^ Department of Infectious Diseases, Singapore General Hospital, Singapore, Singapore; ^5^ Cancer Discovery Hub, National Cancer Centre Singapore, Singapore, Singapore; ^6^ Department of Hepatopancreatobiliary and Transplant Surgery, Singapore General Hospital, Singapore, Singapore; ^7^ National Cancer Centre Singapore, Division of Medical Oncology, Singapore, Singapore; ^8^ Department of Colorectal Surgery, Singapore General Hospital, Singapore, Singapore; ^9^ Cancer Science Institute of Singapore, National University of Singapore, Singapore, Singapore

**Keywords:** case report, SARS-CoV-2, spatial transcriptomics, intra-tumor heterogeneity, tissue-localized immunity, immunotherapy

## Abstract

Severe acute respiratory syndrome coronavirus 2 (SARS-CoV-2) has infected half a billion people, including vulnerable populations such as cancer patients. While increasing evidence supports the persistence of SARS-CoV-2 months after a negative nasopharyngeal swab test, the effects on long-term immune memory and cancer treatment are unclear. In this report, we examined post-COVID-19 tissue-localized immune responses in a hepatocellular carcinoma (HCC) patient and a colorectal cancer (CRC) patient. Using spatial whole-transcriptomic analysis, we demonstrated spatial profiles consistent with a lymphocyte-associated SARS-CoV-2 response (based on two public COVID-19 gene sets) in the tumors and adjacent normal tissues, despite intra-tumor heterogeneity. The use of RNAscope and multiplex immunohistochemistry revealed that the spatial localization of B cells was significantly associated with lymphocyte-associated SARS-CoV-2 responses within the spatial transcriptomic (ST) niches showing the highest levels of virus. Furthermore, single-cell RNA sequencing data obtained from previous (CRC) or new (HCC) *ex vivo* stimulation experiments showed that patient-specific SARS-CoV-2 memory B cells were the main contributors to this positive association. Finally, we evaluated the spatial associations between SARS-CoV-2-induced immunological effects and immunotherapy-related anti-tumor immune responses. Immuno-predictive scores (IMPRES) revealed consistent positive spatial correlations between T cells/cytotoxic lymphocytes and the predicted immune checkpoint blockade (ICB) response, particularly in the HCC tissues. However, the positive spatial correlation between B cells and IMPRES score was restricted to the high-virus ST niche. In addition, tumor immune dysfunction and exclusion (TIDE) analysis revealed marked T cell dysfunction and inflammation, alongside low T cell exclusion and M2 tumor-associated macrophage infiltration. Our results provide *in situ* evidence of SARS-CoV-2-generated persistent immunological memory, which could not only provide tissue protection against reinfection but may also modulate the tumor microenvironment, favoring ICB responsiveness. As the number of cancer patients with COVID-19 comorbidity continues to rise, improved understanding of the long-term immune response induced by SARS-CoV-2 and its impact on cancer treatment is much needed.

## Introduction

The coronavirus disease 2019 (COVID-19) pandemic caused by severe acute respiratory syndrome coronavirus 2 (SARS-CoV-2) has now infected half a billion people, resulting in six million deaths worldwide. As the world enters the third year of the pandemic, research attention has gradually expanded from COVID-19 pathogenesis and treatment to comorbidity management. Cancer patients with compromised immune systems are susceptible to viral infection; thus, cancer represents an important COVID-19 comorbidity (1–4). Increasing evidence supports the prolonged persistence of SARS-CoV-2 in tissues (5–8) and the corresponding long-term immune memory (9, 10), while transcriptional aberrations (11) have been observed months after negative nasopharyngeal swab tests. However, the impact of the *in situ* SARS-CoV-2 immune response on cancer treatment efficacy is unclear.

COVID-19 is closely associated with heightened inflammatory responses. Hence, its post-recovery persistence in the tumor microenvironment (TME) could greatly affect the efficacy of anti-tumor treatment; this finding is particularly relevant for immunotherapies such as immune checkpoint blockade (ICB), which is rapidly emerging as a treatment modality due to its durable response (12, 13). Interestingly, three independent lung cancer cohort studies have counter-intuitively shown that patients with COVID-19 comorbidity who received ICB alone exhibited an equivalent or better response than those receiving other cancer treatments (14). Despite an accumulation of information regarding SARS-CoV-2 immune responses, previous studies did not include cancer patients and were limited to blood analyses (9, 15, 16) and dissociation techniques (10). Furthermore, spatial immune profiling techniques, particularly multiplex immunohistochemistry (mIHC) and digital spatial profiling have demonstrated intra-patient variability in SARS-CoV-2 immune responses across multiple sites (5, 7, 17), underscoring the limitations of blood-based analyses.

In this report, we present spatial whole-transcriptomic profiling analysis of two cancer patients: one with hepatocellular carcinoma (HCC) and one with colorectal cancer (CRC). These patients harbored persistent SARS-CoV-2 in the tissues post-recovery, as we reported previously (5). We evaluated the intra-tissue heterogeneity and immune cell type specificity of the COVID-19 response as well as the potential tissue-localized effects of SARS-CoV-2 persistence on anti-tumor immunity. We aimed to provide additional data on the ICB response in non-lung cancer types. This information will help to lay the foundation for the future development of management strategies for cancer patients who have recovered from COVID-19.

## Case description

A 49-year-old Asian male who was diagnosed with hepatitis B virus (HBV)-positive HCC with no evidence of macrovascular involvement underwent curative segment VII liver resection (3.8 cm × 2.8 cm) 85 days after testing COVID-19 negative using RT-PCR swab test. A second patient, a 45-year-old Asian male diagnosed with invasive stage II T3N0 cecal adenocarcinoma (right-sided CRC), underwent laparoscopic right hemicolectomy 9 days after testing COVID-19 negative using RT-PCR swab test. Patient details have been reported previously (5). The two patients had mild symptoms of acute respiratory infection and were hospitalized for isolation purposes; in addition, they were unvaccinated and did not require oxygen therapy or any medical treatment for COVID-19. Importantly, the patients had not undergone any cancer treatment before COVID-19 infection or surgery, ruling out immunological effects caused by other therapy. While the CRC patient did not have other co-morbidities, the HCC patient had chronic HBV and past TB infections.

Initially, we conducted spatial transcriptomic (ST) profiling on paired specimens consisting of tumor and adjacent normal tissue from the HCC and CRC patients. This analysis was performed on fresh frozen sections using the Visium Spatial Gene Expression (10× Genomics) assay. The tissue sections placed on the capture areas of the Visium slide were fixed, stained with hematoxylin and eosin, and imaged (18). After tissue permeabilization, mRNA molecules were reverse transcribed to generate spatially barcoded cDNA molecules, which were then PCR-amplified and sequenced at 50,000 reads per capture spot. All bioinformatic analysis was performed on individual tissues using the sctransform-normalized data computed by R package Seurat (v4.0.3) (unless stated otherwise) (**Supplementary Figure 1A**).

**Figure 1:**
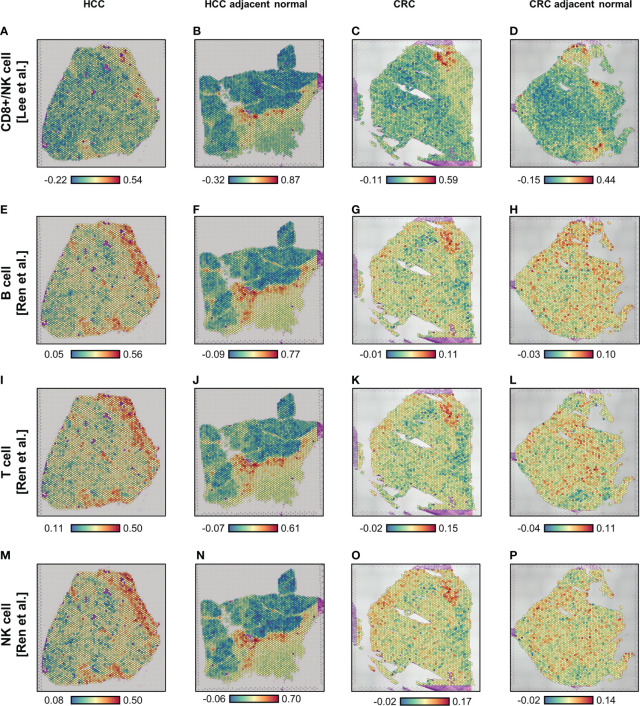
Spatial enrichment of COVID immune response signatures in biopsied HCC and CRC tissues from COVID-19-recovered patients. **(A–P)** Visium-based (10× Genomics) spatial transcriptomics-generated tissue heat maps show the spatial localization of COVID immune responses in HCC **(A, E, I, M)** and CRC **(C, G, K, O)** tumors and their adjacent normal tissues (**B, F, J, N**, **D, H, L, P**, respectively). Analysis was based on lymphocyte-associated COVID immune response gene signatures (Lee et al. (15), **A–D**; and Ren et al. (16), **E–P**): CD8^+^ T/NK cells-associated **(A–D)**, B cell-associated **(E–H)**, T cell-associated **(I–L)**, and NK cell-associated **(M–P)**.

Using the Visium ST data (**Supplementary Figure 2**), we carried out spatial enrichment analysis (*AddModuleScore* function in Seurat) based on the SARS-CoV-2 immune response signatures from two public databases (15, 16) (**Supplementary Table 1**). In the first database, Lee et al. (15) identified lymphocyte-associated (CD8^+^ T cells and NK cells) and myeloid-associated (monocyte and dendritic cells) COVID-19 signatures, using influenza patients as a reference. In the second, Ren et al. (16) determined three lymphocyte-associated (B cell, T cell, and NK cell) COVID-19 signatures and one myeloid-associated (neutrophil) signature, using COVID-19-negative peripheral blood mononuclear cells (PBMCs) as a reference. For the four lymphocyte signatures, spatial profiles were broadly consistent in all four tissues analyzed (**Figure 1**); however, some differences in localization were observed for the SARS-CoV-2 response signatures associated with T cells and NK cells in the CRC-adjacent normal tissue (**Figures 1L, P**). By contrast, myeloid-associated SARS-CoV-2 responses did not demonstrate distinctive spatial localization, except in the HCC-adjacent normal tissue (**Supplementary Figure 3**, see panels **B** and **F**).

**Figure 2:**
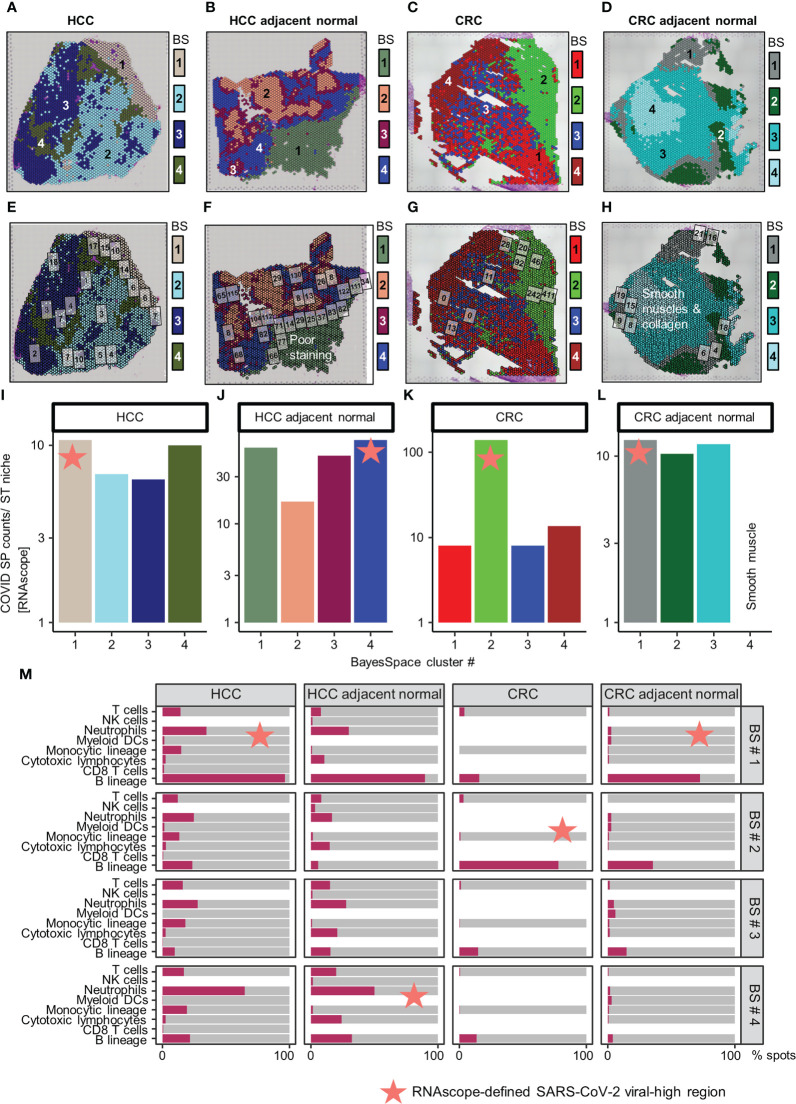
Distribution of SARS-CoV-2 spike protein (SP) and immune cell types in the spatial transcriptomic (ST) niches of HCC and CRC biopsies from COVID-19-recovered patients. **(A–D)** From left to right: HCC, HCC-adjacent normal, CRC, and CRC-adjacent normal tissues; ST niches (clusters 1–4) were determined using the spatially-aware BayesSpace (BS) transcriptomic clustering method. **(E–L)** RNAscope-detected SARS-CoV-2 SP was quantified in regions of interest (marked as boxes) in the Visium-defined ST niches **(E–H)**; see **Supplementary Table 2** for raw data and assignment of ROIs to ST niches. Bar charts **(I–L)** show the relative SP counts in the ST niches; SP counts in ROIs assigned to the same ST niche were averaged. Areas with poor staining quality **(F)** and smooth muscle and collagen **(H, L)** were omitted. **(M)** Distribution of immune cell abundance (estimated by a deconvolution-based microenvironment cell population-counter) in ST niches. The orange stars indicate viral-high regions (ST niches with the highest SARS-CoV-2 SP counts).

Independent of these SARS-CoV-2 immune responses, we identified four regions with distinct ST niches within each tissue using a spatially-aware clustering method (BayesSpace R package v1.2.0) (19) (**Figures 2A–D**); the four clusters were selected arbitrarily to balance resolution and interpretability. Generation of uniform manifold approximation and projection (UMAP) plots was based on the top 30 principal components (*RunPCA* and *RunUMAP* functions in Seurat) of the ST niches (**Supplementary Figure 4**). The distribution of SARS-CoV-2 immune responses was consistent in the ST niches in the HCC tissues but heterogenous in the CRC tissues (**Supplementary Figures 5** and **6**).

As other viral infections (e.g., HBV in the HCC patient) can induce similar immune responses, we further localized SARS-CoV-2 specific immune responses in tissues. This analysis was performed by quantifying SARS-CoV-2 spike protein (SP) counts on serial tissue sections, using RNAscope (**Figures 2E–H**; brightfield images presented in **Supplementary Figures 7**-**8**, raw counts given in **Supplementary Table 2**; **Supplementary Materials**). The SARS-CoV-2 SP counts were spatially variable, especially in the CRC tumor tissue (**Figures 2I–L**). Despite the variability, the viral-high regions (defined as the ST niches exhibiting the highest average SARS-CoV-2 SP counts) were confirmed by mIHC performed on FFPE sections (**Supplementary Figure 9**; **Supplementary Materials**). Both RNAscope and mIHC analyses were based on multiple high-quality regions of interest (ROIs; marked as boxes in **Figures 2E–H** and **Supplementary Figures 9A–D**) that were selected by a pathologist (JY). To compute ST niche-average counts, the images were manually overlaid onto the Visium tissue images, and the ROIs were assigned to the closest ST niches (**Supplementary Table 2**; average SP and NP counts of ROIs assigned to individual ST niches are shown in **Figures 2I–L** and **Supplementary Figures 9E–H**). Furthermore, using a pathologist (JY)-trained machine-learning classifier in QuPath (v0.3.2; Supplementary Materials), the viral-high regions were predominantly identified as stromal or normal epithelial tissues (**Supplementary Figure 10**).

**Figure 3:**
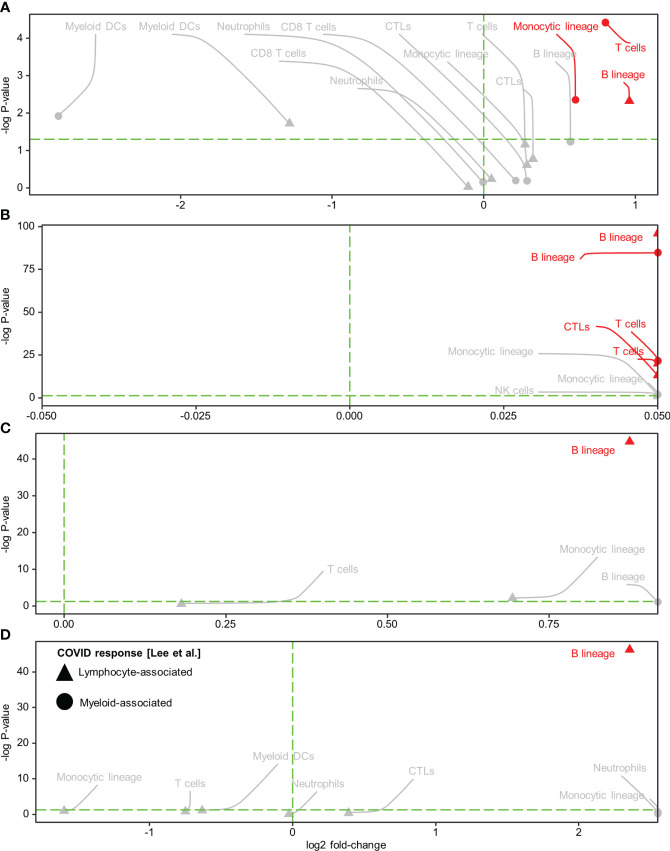
Spatial colocalization of immune cell types and COVID immune response. **(A–D)** Immune cell types (determined by a deconvolution-based microenvironment cell population-counter) and COVID immune responses (quantified as enrichment scores for the COVID response-associated gene signatures reported in Lee et al. (15) within the viral-high regions in HCC **(A)** and CRC **(C)** tumors and the corresponding adjacent normal tissues (**B**, **D**, respectively). Lymphocyte and myeloid-associated COVID responses are represented by triangles and circles, respectively. The vertical green dashed line delineates log-fold change (FC) = 0 (log-FC > 0 for cell types with higher COVID immune scores in Visium spots where the immune cell type was detected than in spots where it was not detected). The horizontal green dashed line represents *P* = 0.05; above the line*, P* < 0.05. Red text and annotations represent *P* < 0.005. *P*-values were computed using the Kruskal-Wallis test. CTLs, cytotoxic lymphocytes; DCs, dendritic cells.

Subsequently, we conducted immune cell type analysis using the microenvironment cell population (MCP)-counter (MCPcounter R package v1.2.0) (20), whereby the presence of a cell type in the Visium spots was defined using a threshold of MCP-counter-estimated log2 expression > 0. The results revealed intra-tissue heterogeneity in terms of tissue coverage (% spots) and detectability (**Figure 2M**). B cells were detected homogeneously within viral-high regions in all tissues (although they were relatively sparse in the HCC-adjacent normal tissue), while other immune cells were either sparse or dispersed throughout the tissue (**Figure 2M**, **Supplementary Figures 11**-**12**). To identify the immune cells that elicited the SARS-CoV-2 immunological effect, we examined the association between the presence of immune cells and public COVID-19 signature scores. Analysis of the COVID-19 signatures identified by Lee et al. (15) showed that within viral-high regions, the lymphocyte-associated SARS-CoV-2 response was significantly positively associated with the spatial localization of B cells across HCC and CRC tissues (*P* < 0.005, **Figures 3A–D**); in contrast, the myeloid COVID-19 signature was only significantly associated with T cells in HCC tissues (*P* < 0.005, **Figures 3A, B**). Similar findings were obtained using the COVID-19 signatures from Ren et al. (16); the lymphocyte-specific SARS-CoV-2 responses (i.e., B, T, and NK-associated responses) were significantly associated with the spatial localization of B cells across HCC and CRC tissues (*P* < 0.005), but not CRC-adjacent normal tissue (**Supplementary Figure 13**).

To further dissect the specificity of B cell subsets involved in the tissue-localized lymphocyte-based COVID-19 response, we characterized and annotated individual B cell phenotypes (memory [MBCs], naïve, intermediate, and plasmablasts) by single-cell analysis of *ex vivo* SARS-CoV-2-stimulated patient-matched samples. Stimulated lymph node samples (CRC) had been obtained previously (5), while PBMCs from the HCC patient were stimulated following the same procedure (5) prior to analysis (Supplementary Materials). Single-cell sequencing used Chromium Single Cell 5’ and 3′ Reagent Kits v2 and v3 (10× Genomics, San Francisco, CA, USA) (21), while paired-end sequencing (2×150 bp) was performed using the NovaSeq 6000 platform (Illumina, Inc., San Diego, CA, USA) (Supplementary Materials; bioinformatic analysis is depicted in **Supplementary Figure 1B**). Marker genes for the B cell phenotypes were identified by comparing SARS-CoV-2-stimulated and unstimulated samples (using the *FindMarkers* function in Seurat; **Supplementary Table 3**). The above-identified patient- and SARS-CoV-2-specific B cell phenotypes were then mapped to individual Visium spots using robust cell type decomposition (RCTD package v1.2.0), whereby the presence of a cell type was recorded if detected as a singlet or a doublet. Within the viral-high regions, plasmablasts and MBCs exhibited the highest spot coverage in HCC and CRC tissues, respectively (**Supplementary Figures 14**-**15**); however, only the spatial localization of MBCs demonstrated a consistently positive association with lymphocyte-associated SARS-CoV-2 immunological effects (**Supplementary Figures 16A–H**). Notably, MBC infiltration (measured by spot coverage) was higher in the viral-high regions of CRC tissues, compared with the viral-high regions of HCC tissues (**Supplementary Figure 14**).

**Figure 4:**
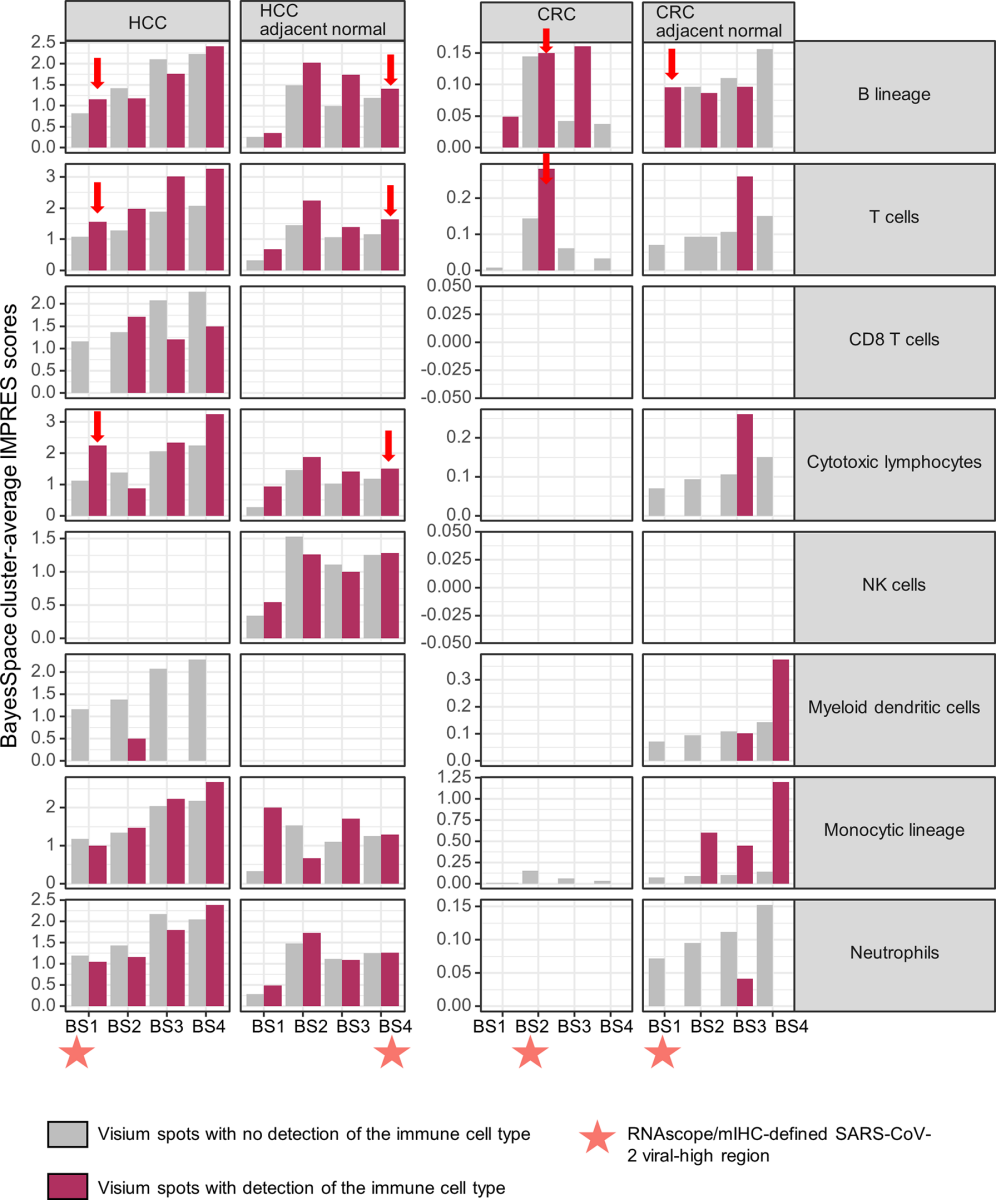
Spatial associations of immune cell types and immuno-predictive scores (IMPRES) in individual spatial transcriptomic niches. From left to right, BayesSpace (BS) clusters 1–4 in HCC, HCC-adjacent normal tissue, CRC, and CRC-adjacent normal tissue are shown. Each BS cluster was stratified by the presence of the immune cell of interest (determined by a deconvolution-based microenvironment cell population-counter) as follows: B lineage, T cells, CD8^+^ T cells, cytotoxic lymphocytes, NK cells, myeloid dendritic cells, monocytic lineage, and neutrophils (from top to bottom). Orange stars indicate viral-high regions (transcriptomic niches with the highest SARS-CoV-2 spike protein/nucleocapsid protein counts); red arrows indicate higher IMPRES scores in Visium spots analyzed for B lineage cells, T cells, and cytotoxic lymphocytes within the viral-high regions.

We evaluated the potential impact of a persistent SARS-CoV-2-induced B cell immune response on spontaneous or immunotherapy-induced anti-tumor activity *in situ* by determining the immuno-predictive scores (IMPRES). IMPRES, a transcriptomic biomarker for ICB, was computed by summing the binary outcomes (gene 1 > gene 2, using spot-level log-normalized counts) of 15 pairs of immune checkpoint genes (**Supplementary Table 4**) and normalizing by the available pairs (22). In individual tissues, the spatial localization of B cells exhibited variable associations (positive and negative) with IMPRES scores across the ST niches. However, within viral-high regions, spots with detection of B cells showed consistently higher IMPRES scores for all four tissues (**Figure 4**; viral-high regions are indicated by orange stars/red arrows). By contrast, spots with detection of T cells and cytotoxic lymphocytes (representing key effector cells in immunotherapy) demonstrated consistently higher IMPRES scores across the ST niches, although they were undetected in some CRC regions. Although the differences in scores were not significant, they were indicative of overall trends. CD8^+^ T cells were generally sparse and not detected in any of the viral-high regions of the four tissues. Other immune cells, including NK cells, myeloid dendritic cells, monocytic lineage cells, and neutrophils, were either not positively associated with IMPRES scores or were undetected in the viral-high regions.

We also evaluated another transcriptomic predictor of ICB, the tumor immune dysfunction and exclusion (TIDE) framework, which simultaneously measures T cell dysfunction and exclusion as key mechanisms of tumor immune evasion. TIDE analysis was conducted on the web platform http://tide.dfci.harvard.edu/ (23), along with the analysis of individual immune components. Specifically, T cell-dysfunction/exclusion, interferon gamma (IFNG), Merck18 (T-cell inflammatory), microsatellite instability (MSI), and tumor-associated macrophages (TAMs; M2 subtype) were analyzed based on ST niche-average log-normalized counts (normalized by log-normalized counts of all other ST niches as a reference). While spatially heterogeneous TIDE scores were observed across the ST niches, TIDE-predicted ICB responsiveness (TIDE scores < 0) was detected in the viral-high regions in both HCC and CRC tumor tissues (**Supplementary Figure 17A**). Notably, compared with other ST niches, the viral-high regions in both HCC and CRC tumor tissues harbored the highest T cell dysfunction, IFNG, and Merck18 signatures (**Supplementary Figure 17B**), alongside the lowest T cell exclusion, microsatellite instability (MSI), and TAM M2 signatures (**Supplementary Figure 17C**). In addition, the viral-high region in HCC tumor tissue harbored the highest expression of the checkpoint molecule CD274 (PD-L1) and the lowest immune cytolytic activity; however, such immune exhaustion was not replicated in the viral-high region of CRC tumor tissue, which might be due to the shorter time elapsed since infection (**Supplementary Figure 18**). Immune cytolytic activity scores were obtained by averaging the *sctransform*-normalized values of two key cytolytic effectors, granzyme A and perforin. Mapping of HBV transcripts (based on 73 HBV variants downloaded from NCBI GenBank; **Supplementary Table 5**) onto ST niches revealed that HBV was detected homogeneously across the HCC tumor tissue but was relatively sparse within the viral-high region; in addition, HBV was not detected in the adjacent normal HCC tissue (**Supplementary Figure 19**).

All analyses were conducted in R (version 4.1.0). Associations were evaluated by the non-parametric Kruskal-Wallis test. Statistical significance was judged using a two-sided α significance level of 0.05.

## Discussion

This case report presents spatial whole-transcriptomic immune analysis of HCC and CRC tissue biopsies that were collected 85 and 9 days, respectively, after the patients had tested negative for COVID-19. These analyses enabled us to investigate the potential impact of long-term immunomodulation induced by tissue-localized SARS-CoV-2 on *in situ* anti-tumor responses. To our knowledge, no similar spatial immune studies have so far been reported for cancer patients with COVID-19 comorbidity.

In distinction to previous studies that were limited to blood analyses (9, 15, 16) and dissociation techniques (10, 11), our analysis revealed intra-tissue heterogeneity in the SARS-CoV-2 immune response. In addition, the results suggested a robust lymphocyte-associated SARS-CoV-2 post-recovery response, given that the spatial profiles for lymphocyte-associated SARS-CoV-2 responses (based on gene signatures from two independent public databases) were largely comparable. In contrast, the spatial profiles relating to myeloid-associated SARS-CoV-2 responses were weak and heterogeneous. Of note, the gene signatures reported in Ren et al. (16) were obtained specifically from patients with highly active COVID-19; thus, our data indicated that tissue-localized immune responses remained active in the patients in our study following recovery from the virus.

Leveraging the spatial information afforded by the 10× Visium technology, we investigated the immune cells involved in this persistent tissue-localized lymphocyte-based SARS-CoV-2 response. Within viral-high regions, B cells showed consistently positive associations with the lymphocyte-mediated SARS-CoV-2 response in both HCC and CRC tissues. Data obtained from *ex vivo* SARS-CoV-2 peptide stimulation provided statistically significant evidence of the key role played by a specific B cell subset (i.e., MBCs) in eliciting these SARS-CoV-2-specific lymphocyte responses. These findings support previous reports that SARS-CoV-2 infection generates persistent circulating (8) and tissue-localized MBCs (10), detectable by flow cytometry 6 months after infection. In addition, comparison of the 9-day (CRC) and 85-day (HCC) post-COVID-19 data suggested a potential decrease in MBC-associated SARS-CoV-2 protection with length of time since the negative swab test.

Apart from the observation that SARS-CoV-2 infection generates MBCs that protect against viral reinfection, our analysis suggests that these cells also change the anti-tumor properties of the TME. We demonstrated that, despite consistently higher IMPRES scores (predictive of ICB response) within regions containing T cells and cytotoxic lymphocytes (key effector cells in ICB treatment), the spatial localization of B cells was associated with higher IMPRES scores only within the viral-high regions. These findings imply that the SARS-CoV-2-specific B cell response may modulate the TME in favor of ICB responsiveness. Moreover, they might potentially explain, in part, the better performance of cancer patients in response to ICBs alone than those treated with other cancer treatments (14). Indeed, it has been suggested that B cell-mediated tumor killing is part of the ICB response (24). Potential ICB responsiveness within the viral-high regions in both HCC and CRC tumor tissues was confirmed *in silico* using TIDE as another predictor of ICB. Spatially resolved analysis of TIDE component scores further revealed that ICB responsiveness could be attributed to highly infiltrated T cells (i.e., low T cell exclusion) that were dysfunctional (i.e., high T cell dysfunction) but rescuable by ICB. Furthermore, IFNG and T cell-inflamed (Merck18) signatures were enriched within the viral-high regions, while immunosuppressive M2 TAMs were depleted. Interestingly, MSI transcriptomic scores were relatively low within these viral-high regions, suggesting that high T cell infiltration was driven by viral infection, especially SARS-CoV-2 or HBV, rather than by mutation. These data, i.e., exhaustion of B cells and depletion of T cells within the viral-high regions, may explain the long-term co-existence of SARS-CoV-2 and its associated B cells in HCC tissues. Moreover, the rather depleted levels of HBV in the viral-high regions of HCC tissue and the total absence of HBV in adjacent normal tissue suggested that the immune exhaustion and decreased immune cytolytic activity was driven mainly by SARS-CoV-2, with limited influence exerted by HBV.

Due to difficulties obtaining post-COVID-19 tissues (especially treatment-naïve tissues, to rule out treatment effects), we could only include single patients for HCC and CRC. However, the consistency of our findings in two different tumor types suggests that these effects were unlikely to be patient-specific. Although the HCC patient had co-morbidities of HBV and TB, HBV was relatively depleted in the SARS-CoV-2-high tissue region, while the associated B-cell response was consistent with that identified in the CRC patient (who lacked co-morbidities). Taken together with the SARS-CoV-2 stimulation results, these data support the hypothesis that persistent B-cell immune response and any potential effects on ICB therapy are highly likely to be specific to SARS-CoV-2. Moreover, while intra-tumor heterogeneity is recognized as a clinically significant aspect of a comprehensive TME assessment, there is currently no consensus on resolution level. We applied the spatially-aware BayesSpace clustering method to define four micro-TME regions in each of the tissues, selected arbitrarily to ensure a balance between resolution and interpretability. However, sensitivity analysis should be performed on the size and resolution of the micro-TMEs when a larger sample size is available. In addition, as the COVID-19 cases we examined occurred in the pre-vaccine era, our study did not account for the effect of vaccine-induced MBCs. Finally, the degree of persistence of SARS-CoV-2-induced immune responses in long COVID cases (patients exhibiting long-term persistence of COVID symptoms) requires further investigation, using tissues collected at different times after recovery. Nonetheless, we have demonstrated a nuanced view of the persistent *in situ* immunological effects of SARS-CoV-2 infection. These findings provide a foundation to evaluate the observed B cell-mediated immunomodulation of the TME, which appears to favor ICB responsiveness. Furthermore, our results underscore the importance of developing strategies for the management of the increasing number of cancer patients with COVID-19 comorbidity.

## Data availability statement

The datasets presented in this study can be found in online repositories. The names of the repository/repositories and accession number(s) can be found below: NCBI under accession ID PRJNA858545.

## Ethics statement

This study was approved by the SingHealth Centralized Institutional Review Board (reference numbers: 2018/3045 and 2019/2653).

## Author contributions

Conception, design, and supervision: JinL, TL, and JY. Drafting of the article: ML, YY, DG, CChe, BT, JefL, FW, XL, and CL. Bioinformatics and data analysis: ML, YY, BT, XL, and CL. Provision of patient information: WL, PC, CCha, YK, TT, SK, WT, JN, and JenL. Assistance with histology-related techniques: JefL, CJ, JuL, and HB. Performed Visium experiment: JefL, JiL, and CN. Performed single-cell sequencing and stimulation experiment: DG and XL. Generated figures: ML, BT, and CL. All authors have read and agreed to the published version of the manuscript.

## Funding

The authors received funding from the Centre Grant of Singapore General Hospital (grant no. NMRC/CG/M011/2017_SGH) and an A*STAR Career Development Award (C210112056).

## Conflict of interest

The authors declare that the research was conducted in the absence of any commercial or financial relationships that could be construed as a potential conflict of interest.

## Publisher’s note

All claims expressed in this article are solely those of the authors and do not necessarily represent those of their affiliated organizations, or those of the publisher, the editors and the reviewers. Any product that may be evaluated in this article, or claim that may be made by its manufacturer, is not guaranteed or endorsed by the publisher.
