# Supplementary material for: Case report: Understanding the impact of persistent tissue-localization of SARS-CoV-2 on immune response activity via spatial transcriptomic analysis of two cancer patients with COVID-19 co-morbidity
Source: Front Immunol. 2022 Sep 12;13:978760. doi: 10.3389/fimmu.2022.978760 (PMC9510984; doi:10.3389/fimmu.2022.978760)
Supplement: Supplementary file 1 [file Presentation_1.pdf]

Supplementary Materials

Machine learning-based tissue categorization

Using the Visium companion H&E images (i.e., H&E staining on the same tissue sections used for Visium), a pathologist (JY) trained a machine-learning classifier using QuPath (v0.3.2) to categorize each tissue as tumor or normal epithelium, stroma, and excluded regions with tissue damage and high collagen/smooth muscle, by providing manually annotated training tissue regions.

Ex vivo SARS-CoV-2 peptide stimulation

Cells isolated from the HCC (PBMCs) patient underwent ex vivo peptide stimulation following the same procedure for CRC (lymph nodes, LNs) patient as reported previously [1]. We used SARS-CoV-2 PepTivator Peptide Pools (Miltenyi Biotec, Germany) containing the spike protein (PepTivator® SARS-CoV-2 Prot_S), the membrane glycoprotein (PepTivator® SARS-CoV-2 Prot_M), and the nucleocapsid phosphoprotein (PepTivator® SARS-CoV-2 Prot_N). Lyophilised peptide pools were reconstituted as per manufacturer's instructions. 1×106 cells were stimulated with 1 µg/mL peptides for 16 hours at 37°C in 5% CO2 in RPMI 1640 media (Gibco, USA) supplemented with 10% FBS (Hyclone) and 1% Penicillin-Streptomycin-Glutamine (Gibco, USA). Negative controls were left unstimulated. Brefeldin A (1 µg/ml, Sigma Aldrich, Germany) was added 2 hours into the stimulation assay.

CRC tissue dissociation and cell isolation

As reported previously [1], CRC patient-matched lymph nodes were cut into fine pieces and digested with 0.2 mg/mL Collagenase IV (Gibco; Thermo Fisher Scientific, Inc, Waltham, MA, USA) and 0.5 mg/mL DNAse I (Sigma-Aldrich; Merck KGaA, Darmstadt, Germany) in complete RPMI 1640 (Gibco; Thermo Fisher Scientific, Inc.) for 20 min at 37 °C. Digested

tissue was filtered using a 70-μm cell strainer, and the cells were treated with ACK lysing buffer (Gibco; Thermo Fisher Scientific, Inc.) for 5 min at room temperature. Cell debris was removed using Debris Removal Solution according to the manufacturer's protocol (Miltenyi Biotec, Ltd., Bergisch Gladbach, Germany).

10x Genomics Chromium single-cell RNA sequencing (scRNA-seq)

About 16,000 cells were loaded into the Chromium Controller for a targeted recovery of 10,000 single cells. The cells were partitioned into nanoliter-scale Gel Bead-In Emulsions and individually barcoded. The 10x Genomics Chromium Single Cell 5' and 3' Reagent Kits v2 and v3 (10x Genomics, San Francisco, CA, USA) were used for reverse transcription, cDNA amplification, and library construction of gene expression libraries according to the manufacturer's instructions. Library quality was assessed using BioAnalyzer 2100 with Agilent High Sensitivity DNA Kit (Agilent Technologies, Inc., Santa Clara, CA, USA). Paired-end sequencing at 2 × 150 bp was performed using the Illumina Novaseq 6000 platform (Illumina, Inc., San Diego, CA, USA).

Cell-type annotations with robust cell type decomposition (RCTD)

The RCTD package (v1.2.0) was used for patient- and-COVID-specific immune cell annotation of each Visium spot. Using ex vivo stimulated single-cell data as the reference, cell-types were first mapped from the multimodal peripheral blood mononuclear cells (PBMC) reference dataset[2]. The presence of a cell type was recorded if detected as a singlet or a doublet. Marker genes for B cells (memory (MBCs), naïve, intermediate and plasmablasts) were identified by comparing SARS-CoV-2 stimulated against unstimulated samples (*FindMarkers* function in Seurat) (Supplementary Table 1).

RNAscope assay and mIHC

In situ hybridisation was performed using an RNAscope 2.5 HD Duplex Reagent Kit (Advanced Cell Diagnostics, CA, USA; Cat# 322430) where deparaffinised and rehydrated FFPE tissues were subjected to peroxidase inhibition, pre-treatment, incubation with the SARS-CoV2 Spike probe (SP; Cat# 848561) and haematoxylin counterstain [3 4]. Images were acquired using an Axio Scan Z1 (Carl Zeiss, Germany).

mIHC was performed on FFPE sections using a Leica Bond Max autostainer (Leica Biosystems, Melbourne, Australia) [1]. SARS-CoV-2 nucleocapsid protein (NP; Novus Biologicals, CO, USA; Cat# NB100-56576) expression was detected using an Opal 6-Plex Detection Kit (Akoya Biosciences, CA, USA) and imaged using a Vectra 3 pathology imaging system (Akoya Biosciences).

References

1. Cheung CCL, Goh D, Lim X, Tien TZ. Residual SARS-CoV-2 viral antigens detected in GI and hepatic tissues from five recovered patients with COVID-19. *Gut* 2021:gutjnl-2021-324280. doi: 10.1136/gutjnl-2021-324280
2. Hao Y, Hao S, Andersen-Nissen E, Mauck WM, 3rd. Integrated analysis of multimodal single-cell data. *Cell* 2021;184(13):3573-87.e29. doi: 10.1016/j.cell.2021.04.048 [published Online First: 2021/06/02]
3. Wang F, Flanagan J, Su N, Wang L-C. RNAscope: a novel in situ RNA analysis platform for formalin-fixed, paraffin-embedded tissues. *The Journal of molecular diagnostics* 2012;14(1):22-29.
4. Chandrashekar A, Liu J, Martinot AJ, McMahan K. SARS-CoV-2 infection protects against rechallenge in rhesus macaques. *Science* 2020;369(6505):812-17.
