# Supplementary material for: Case report: Understanding the impact of persistent tissue-localization of SARS-CoV-2 on immune response activity via spatial transcriptomic analysis of two cancer patients with COVID-19 co-morbidity
Source: Front Immunol. 2022 Sep 12;13:978760. doi: 10.3389/fimmu.2022.978760 (PMC9510984; doi:10.3389/fimmu.2022.978760)
Supplement: Supplementary file 2 [file Presentation_2.pdf]

A **Spatial transcriptomic data preprocessing**

Space Ranger (10× Genomics) : aligned reads/UMIs to the GRCh38 human reference genome (GenBank Assembly ID GCA_000001405.28) ← *Fastq files, H&E images tif files*; ← *JSON files*

Loupe Browser (10× Genomics) : located spatial barcodes to tissue images

↓

Seurat R package (v4.0.3): cleaned data by removing Visium spots containing (i) <200 (HCC) or <50 (CRC) unique genes, (ii) >6,500 unique genes (doublets), or (iii) >20% (HCC) or >25% (CRC) mitochondrial genes (low-quality or dying cells)

↓

Seurat R package (v4.0.3): (i) identified 3,000 most variable genes, and (ii) computed sctransform-normalized data (*sctransform* function)

B **Single-cell transcriptomic data preprocessing**

Cell Ranger (10× Genomics) : aligned reads/UMIs to the GRCh38 human reference genome (GenBank Assembly ID GCA_000001405.28) ← *Fastq files*

↓

Seurat R package (v4.0.3): cleaned data by removing cells containing (i) <200 unique genes (low-quality cells or empty droplets), (ii) >4,000 unique genes (doublets), or (iii) >35% mitochondrial genes (low-quality or dying cells)

↓

Seurat R package (v4.0.3): (i) identified 3,000 most variable genes, and (ii) computed sctransform-normalized data (*sctransform* function)

Supplementary Figure 1

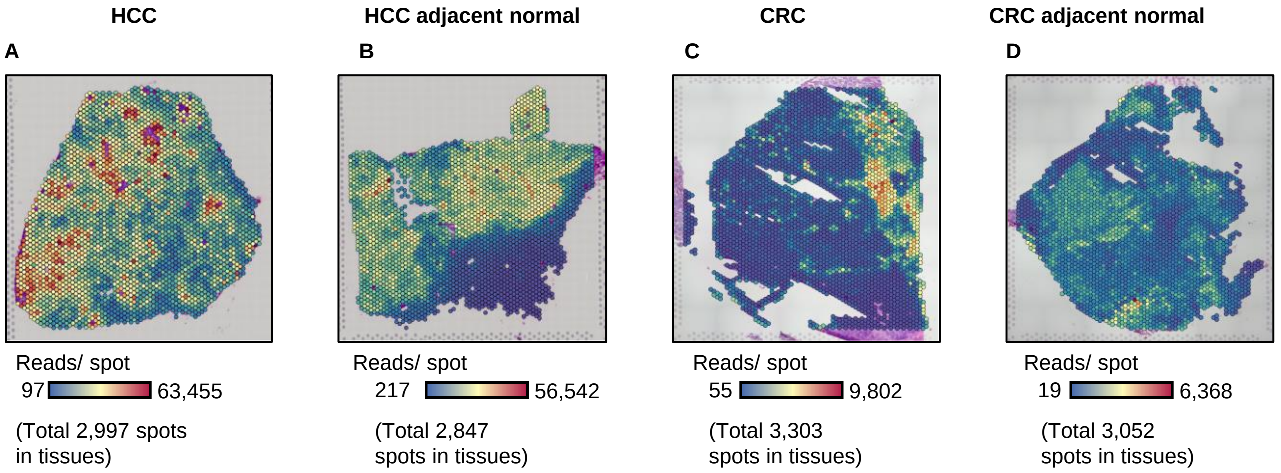

Supplementary Figure 2

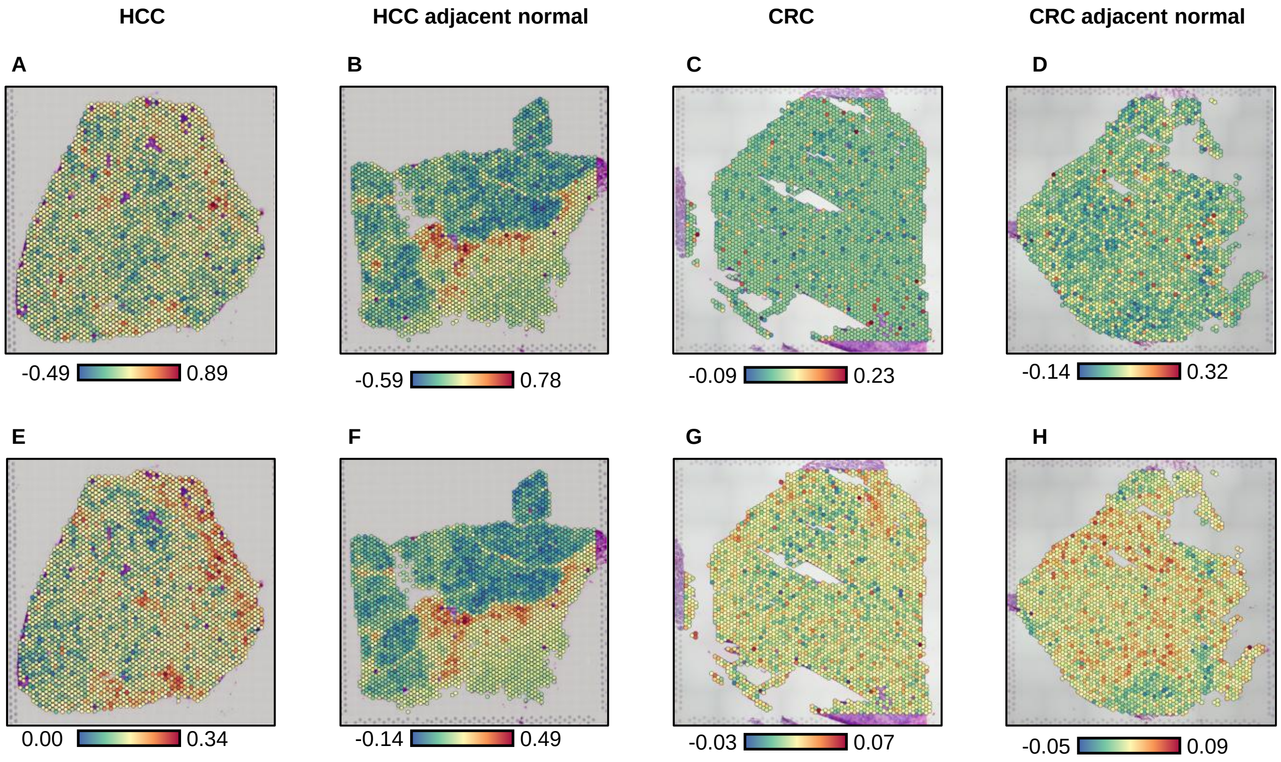

Supplementary Figure 3

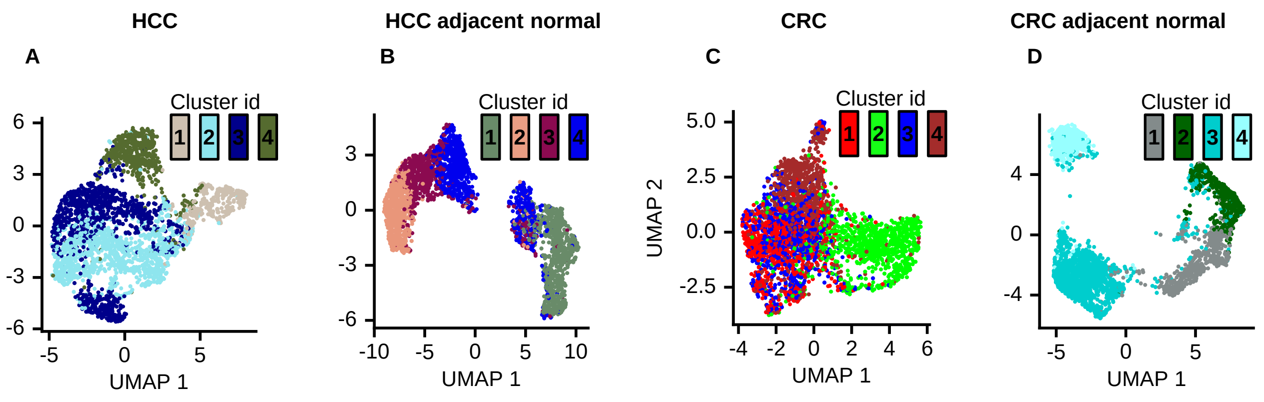

Supplementary Figure 4

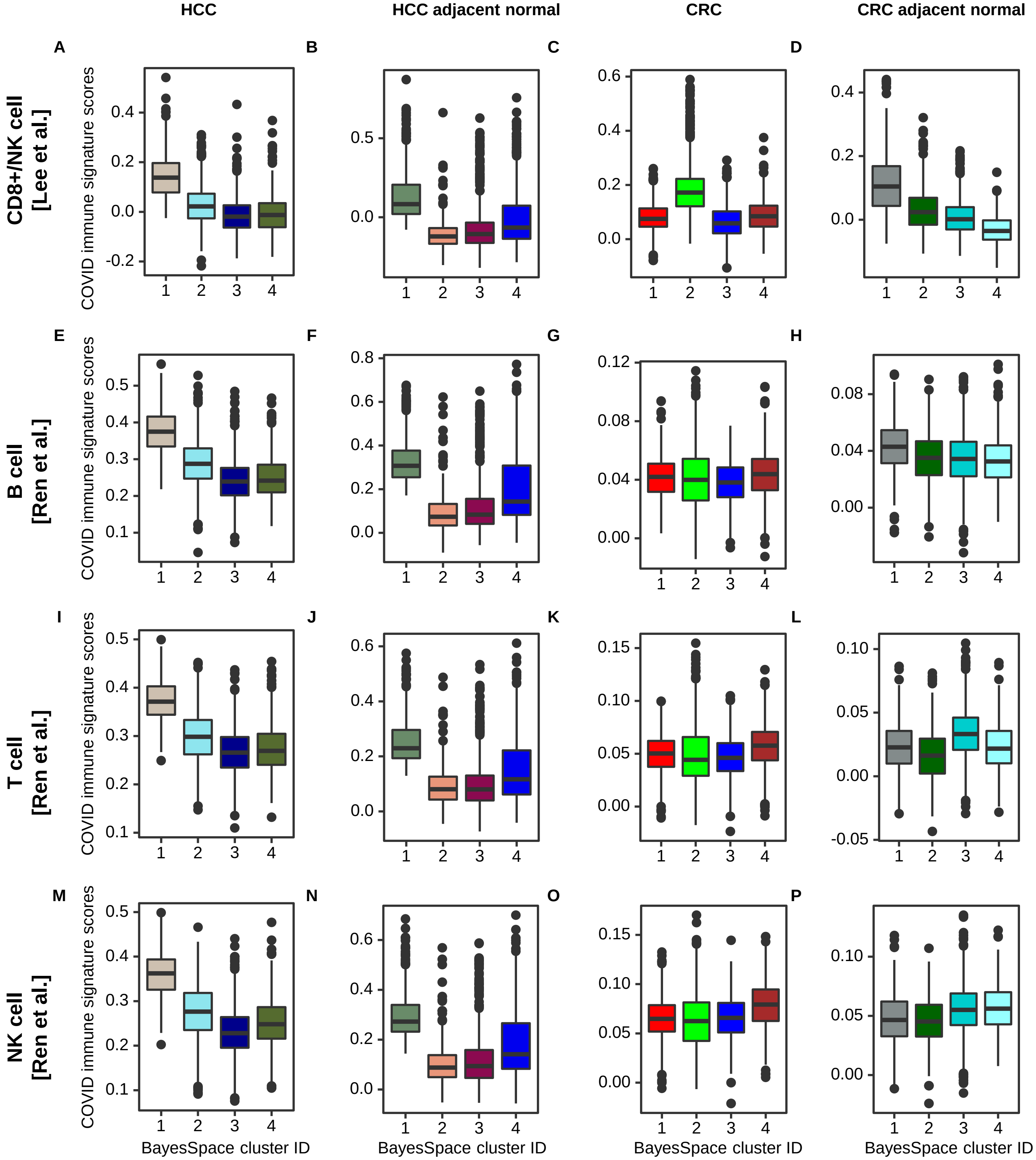

Supplementary Figure 5

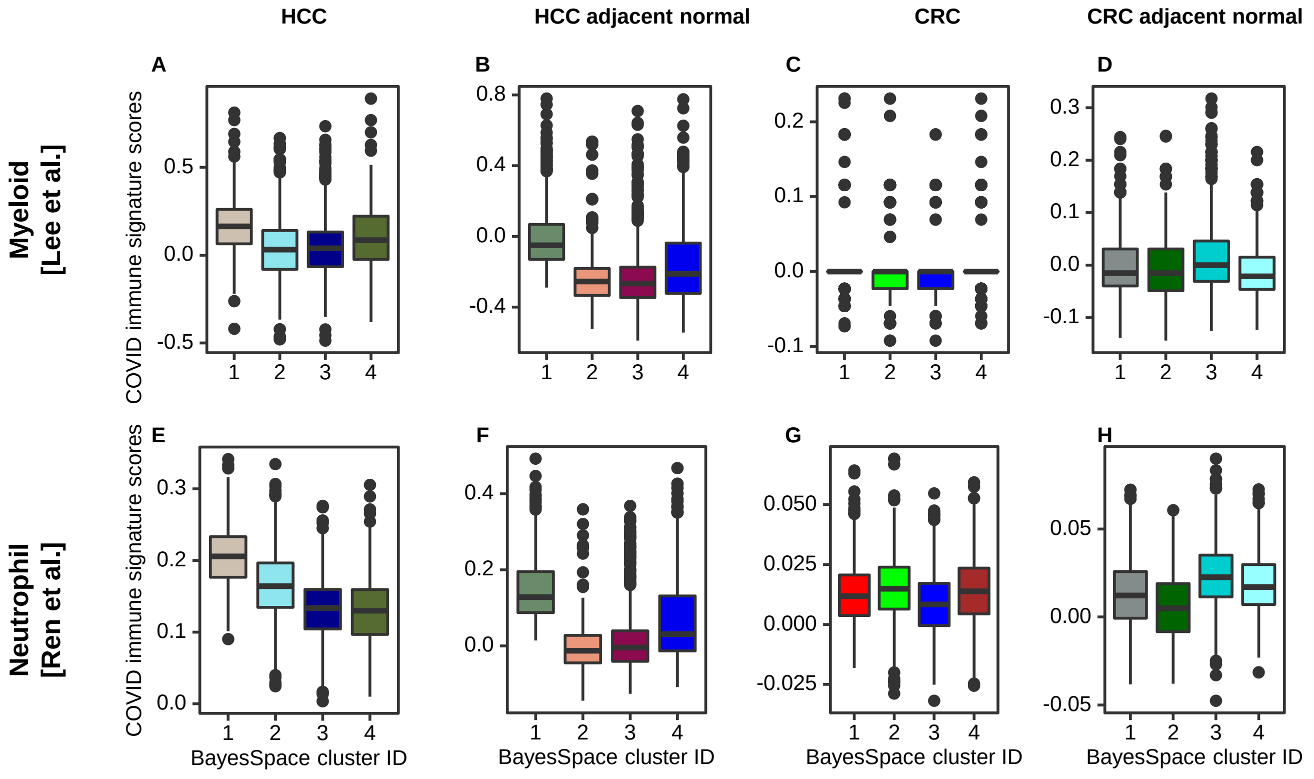

Supplementary Figure 6

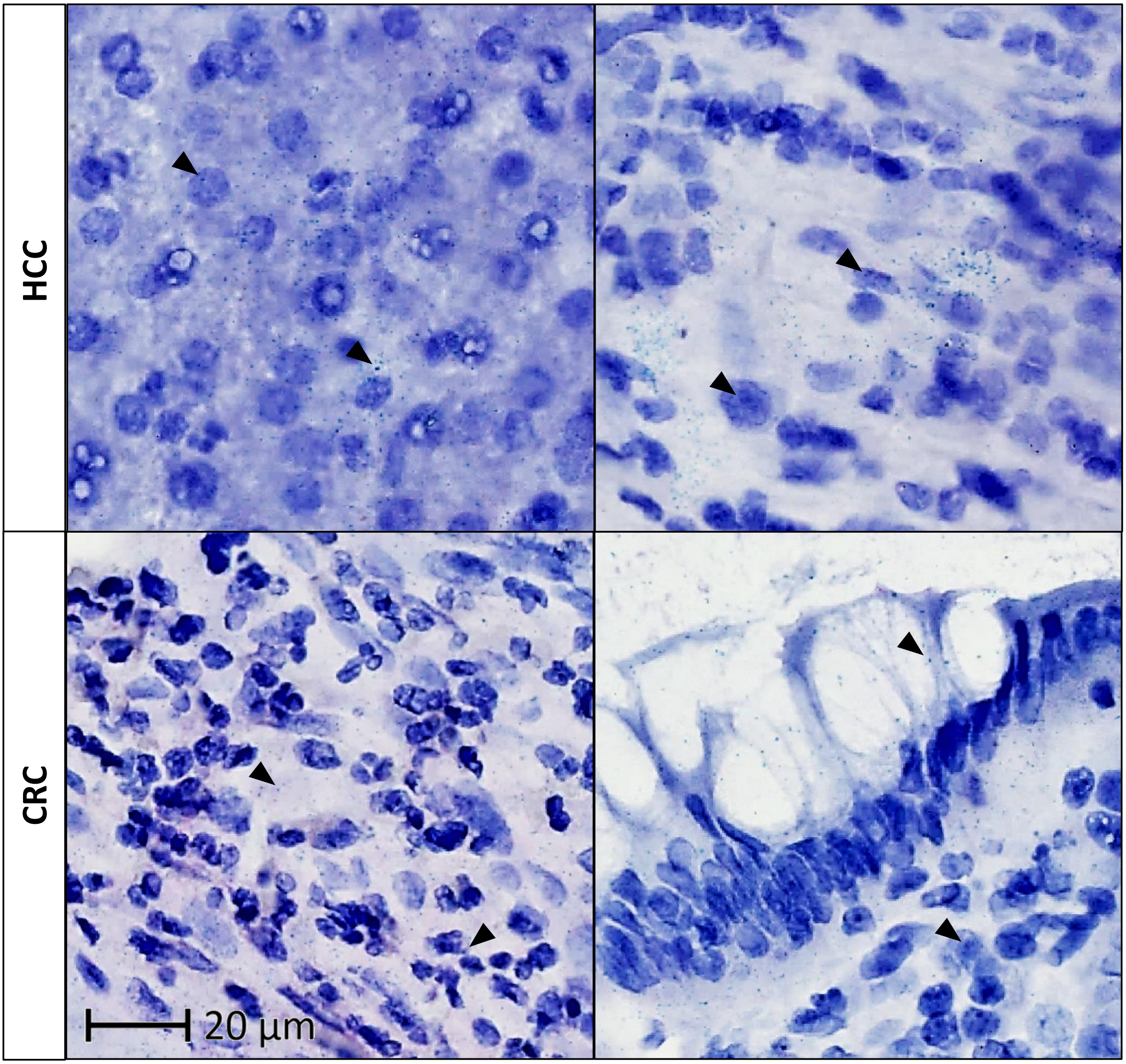

Supplementary Figure 7

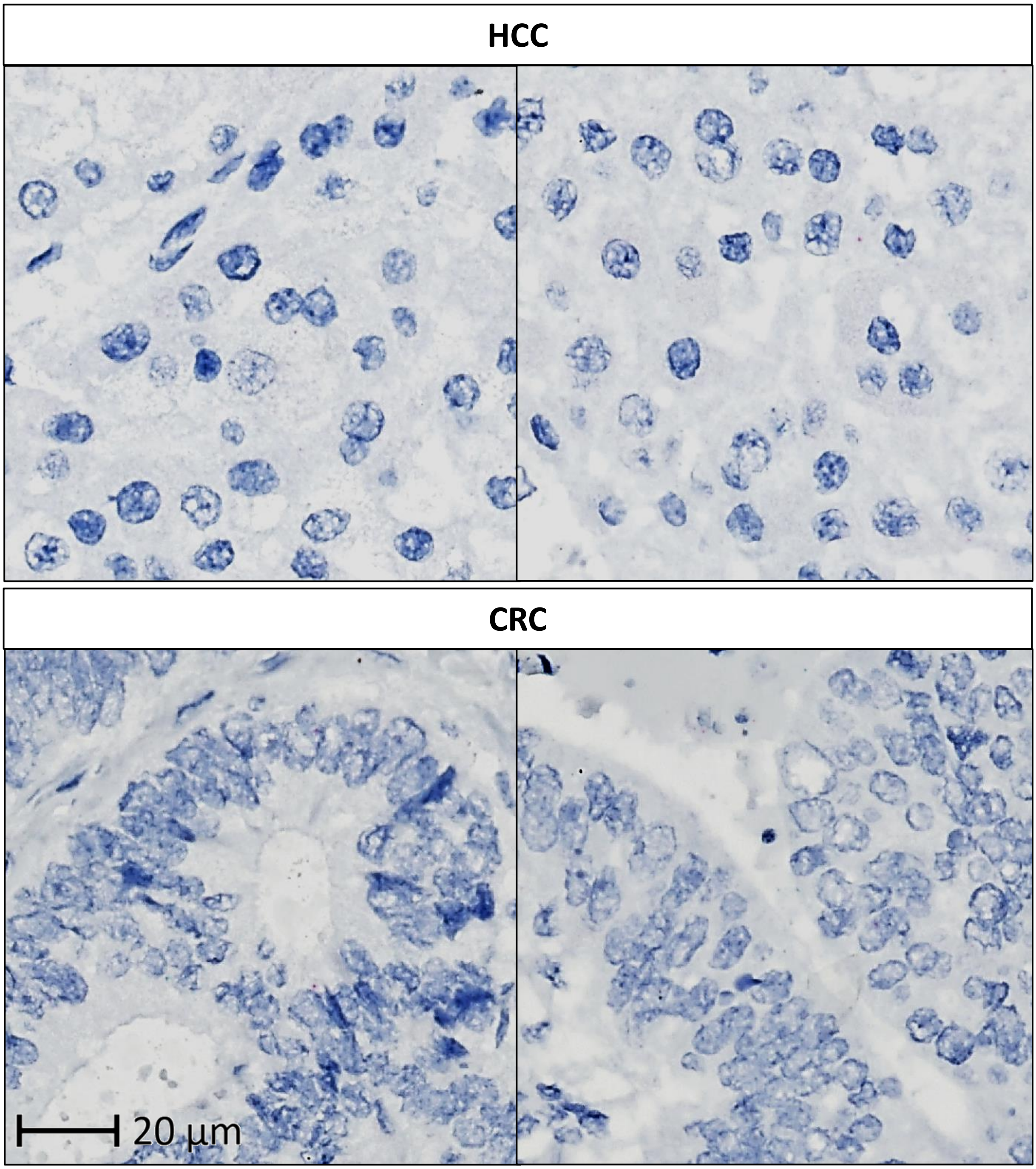

Supplementary Figure 8

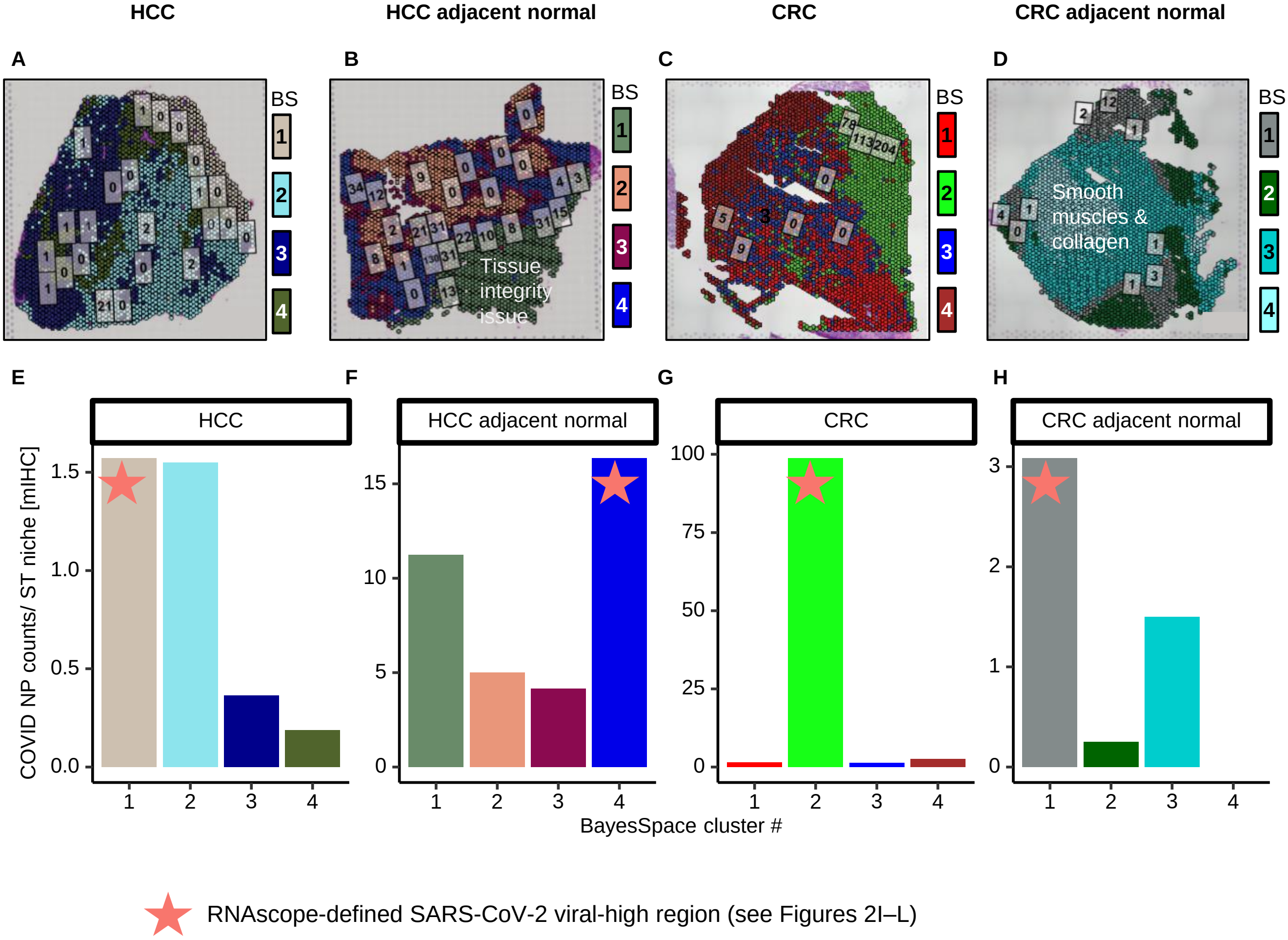

Supplementary Figure 9

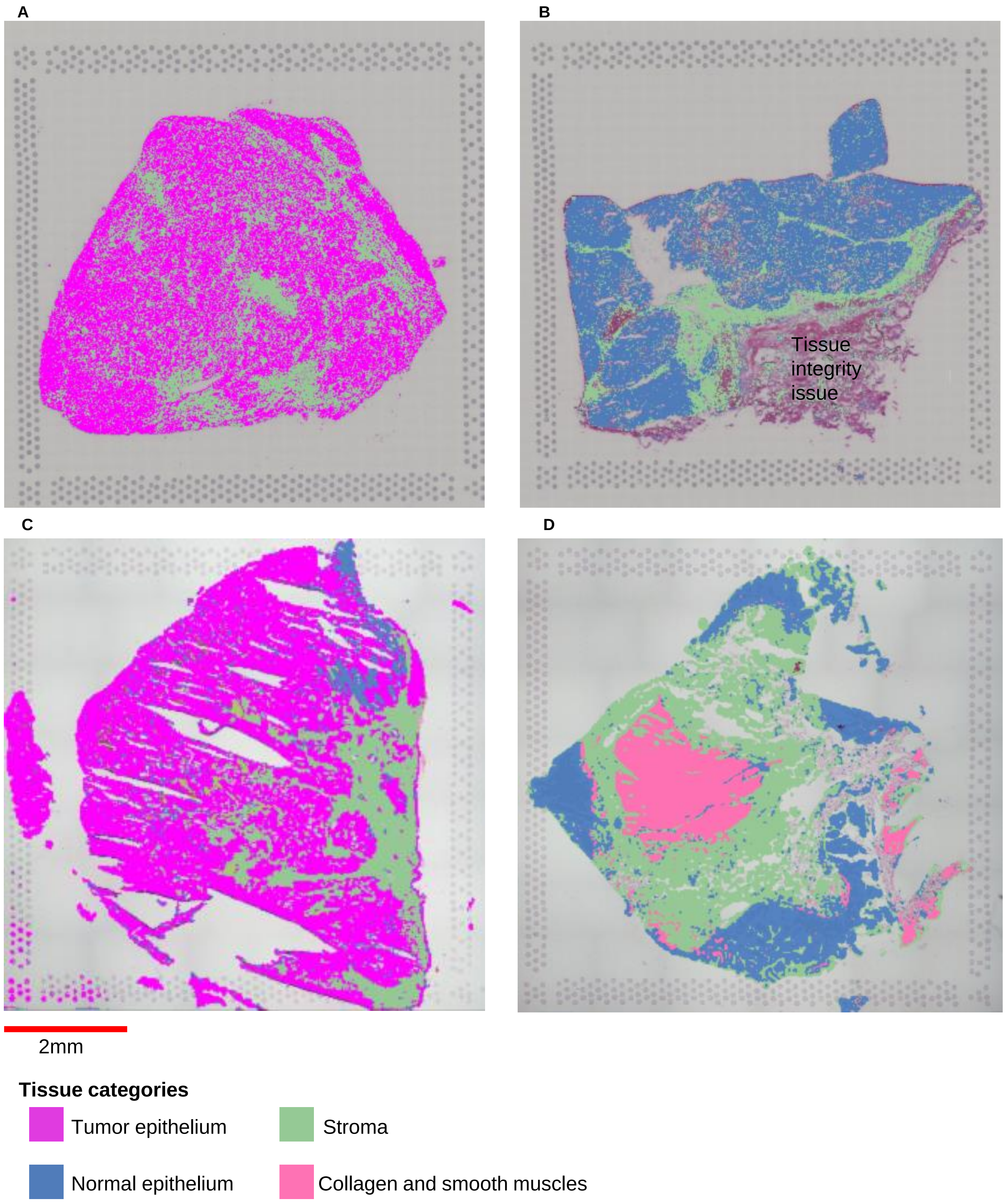

Supplementary Figure 10

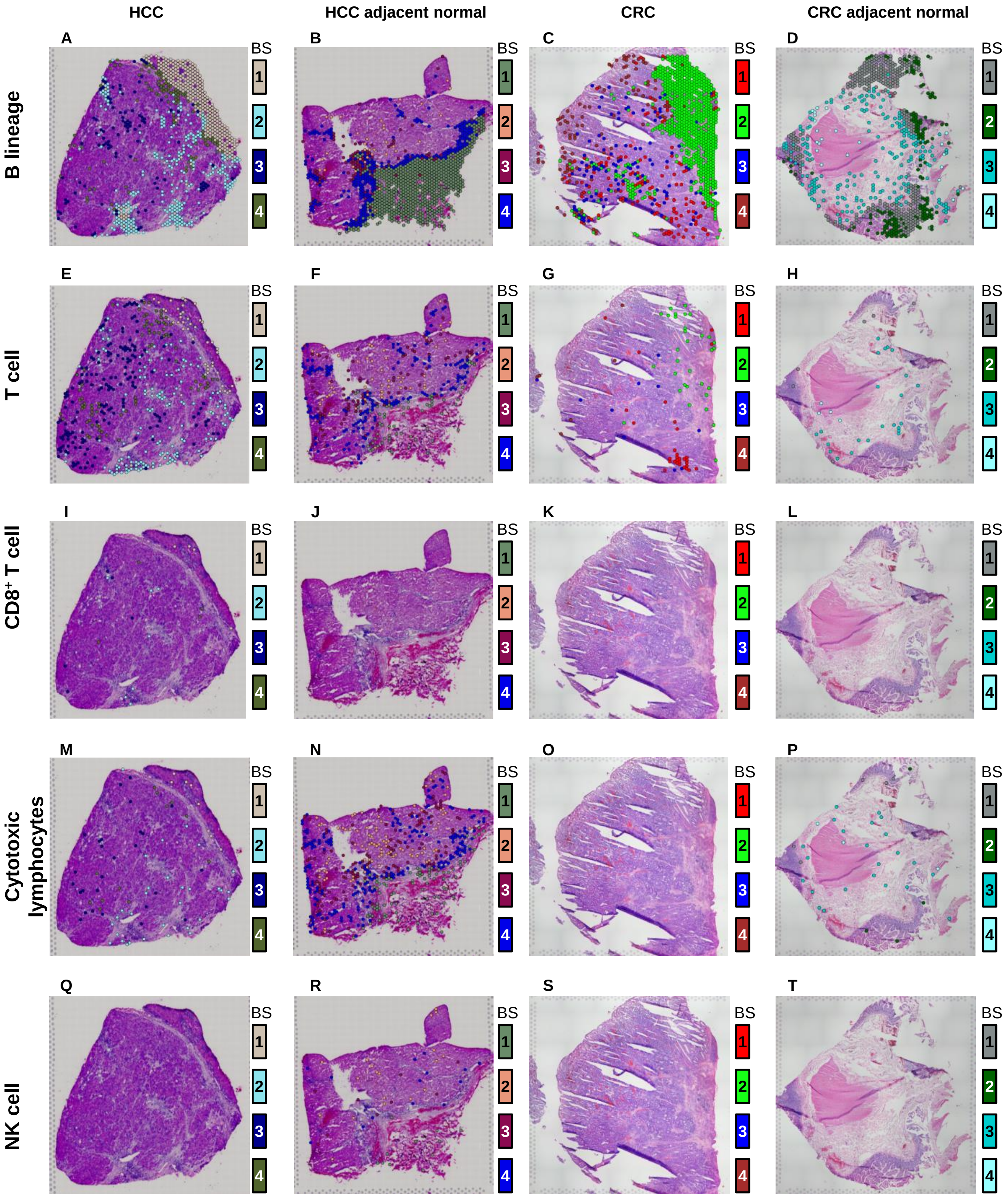

Supplementary Figure 11

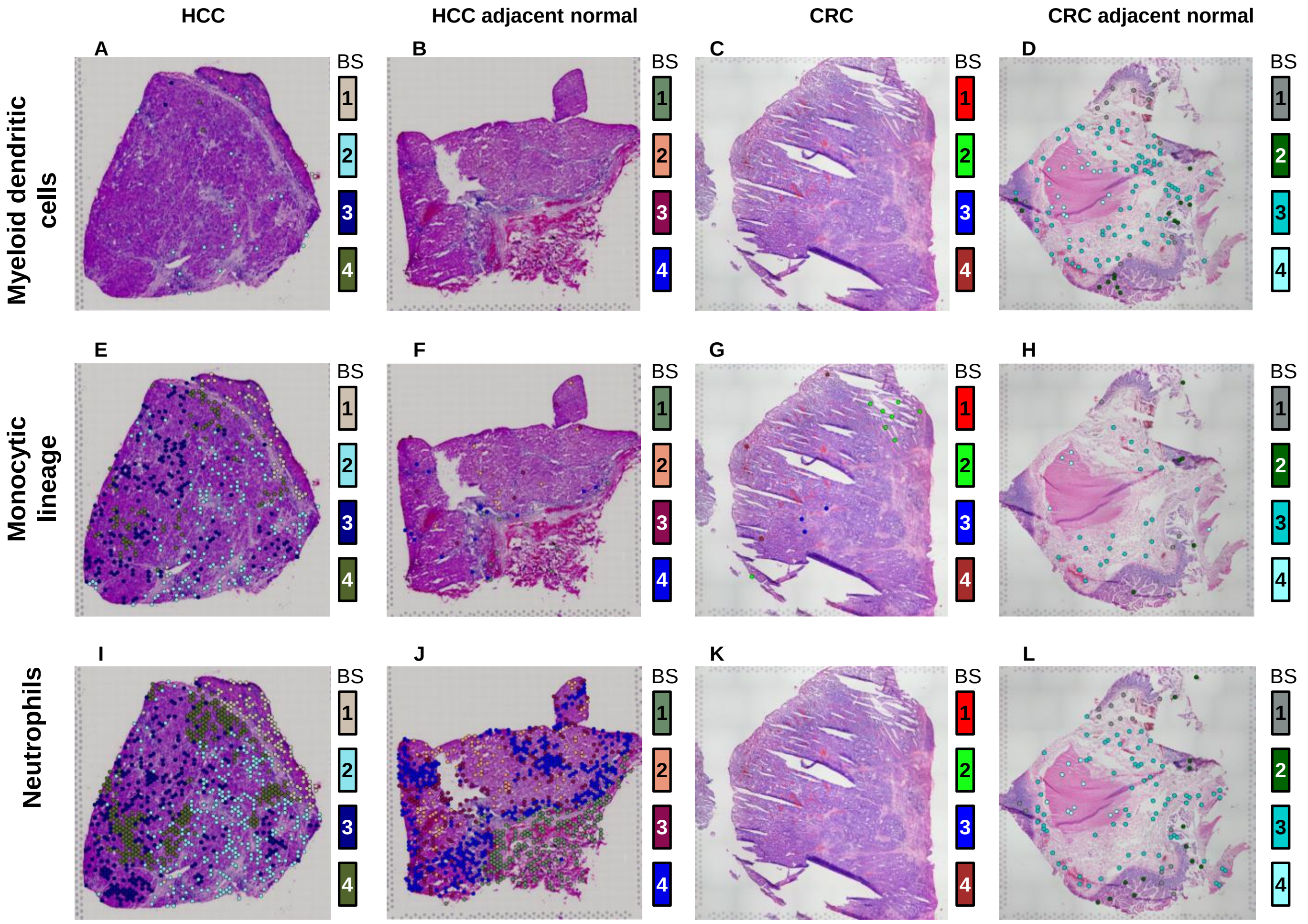

Supplementary Figure 12

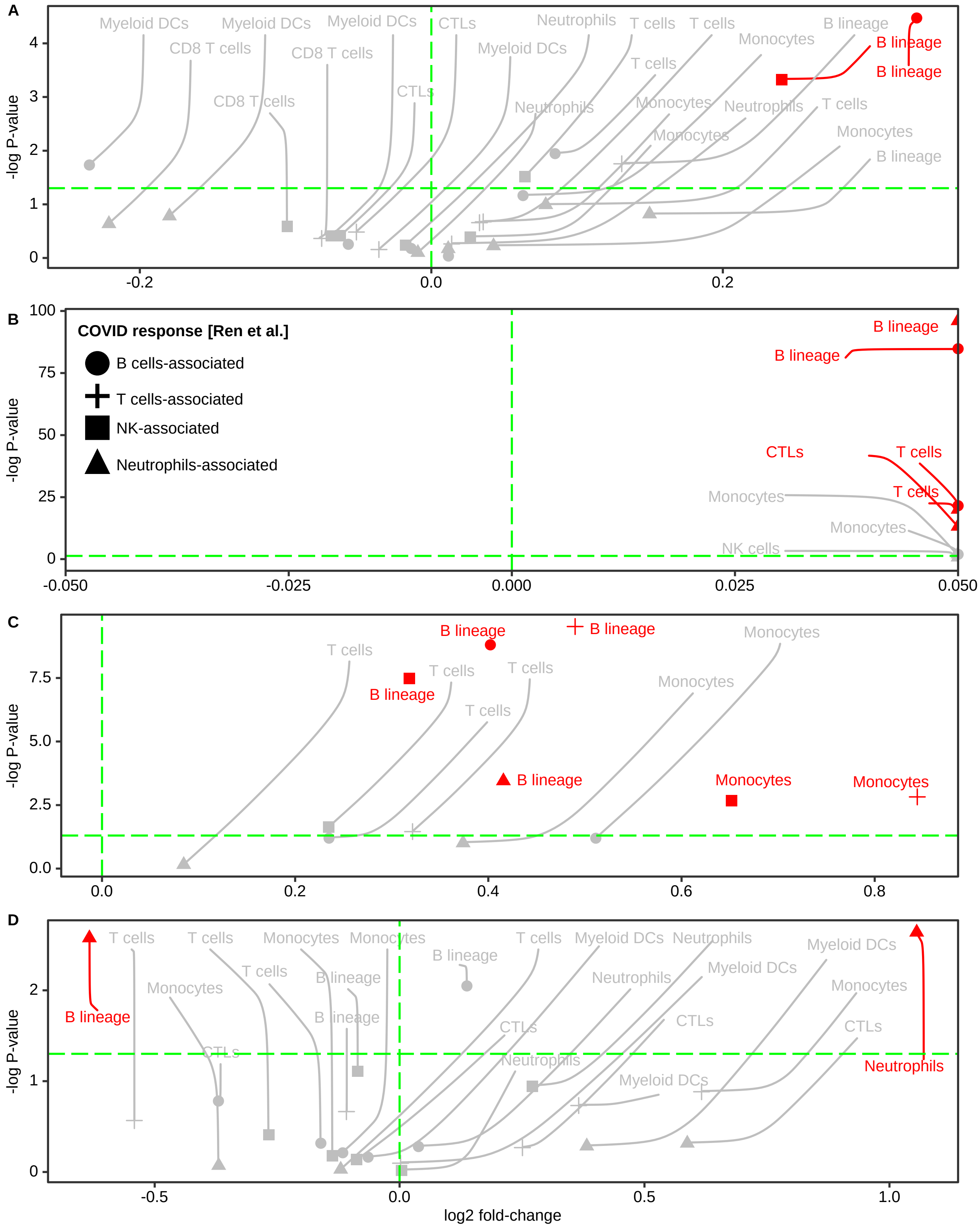

Supplementary Figure 13

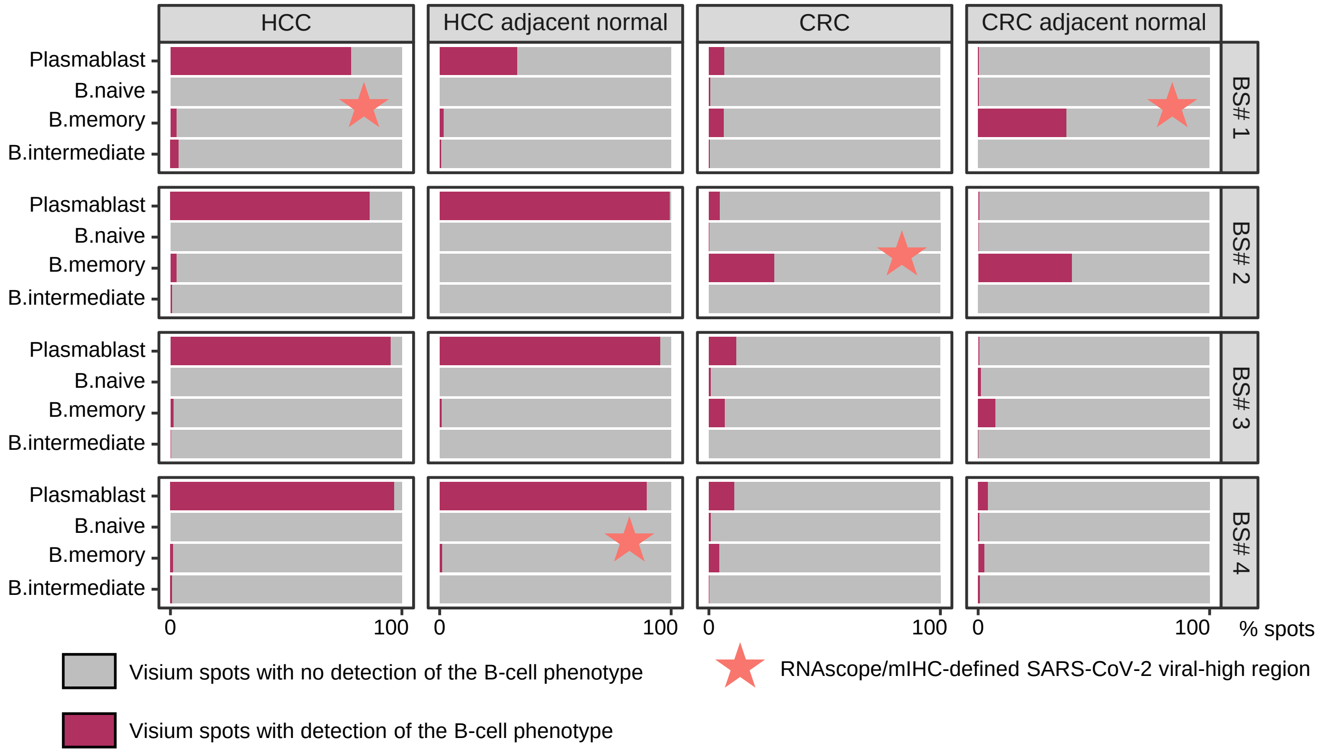

Supplementary Figure 14

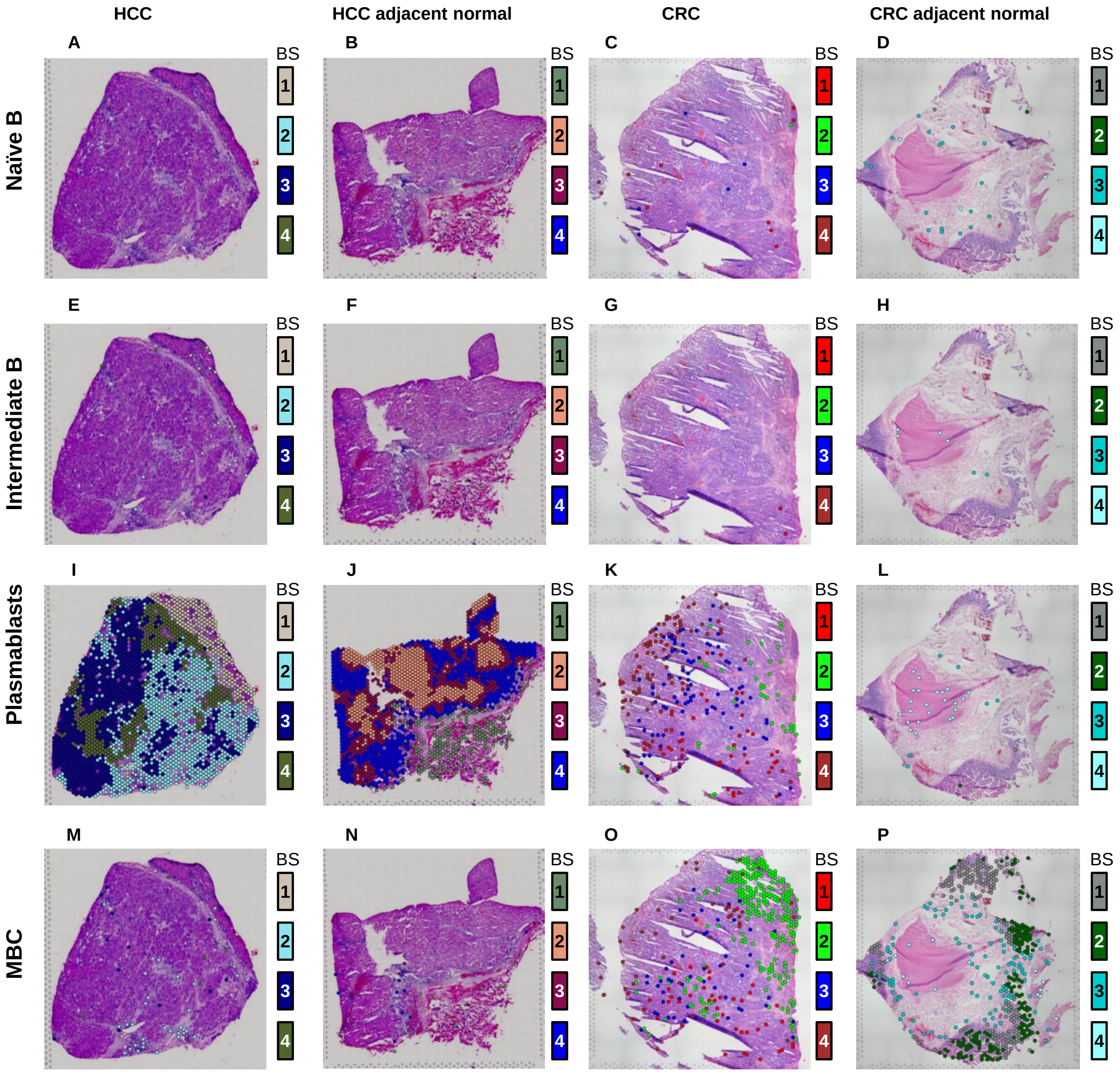

Supplementary Figure 15

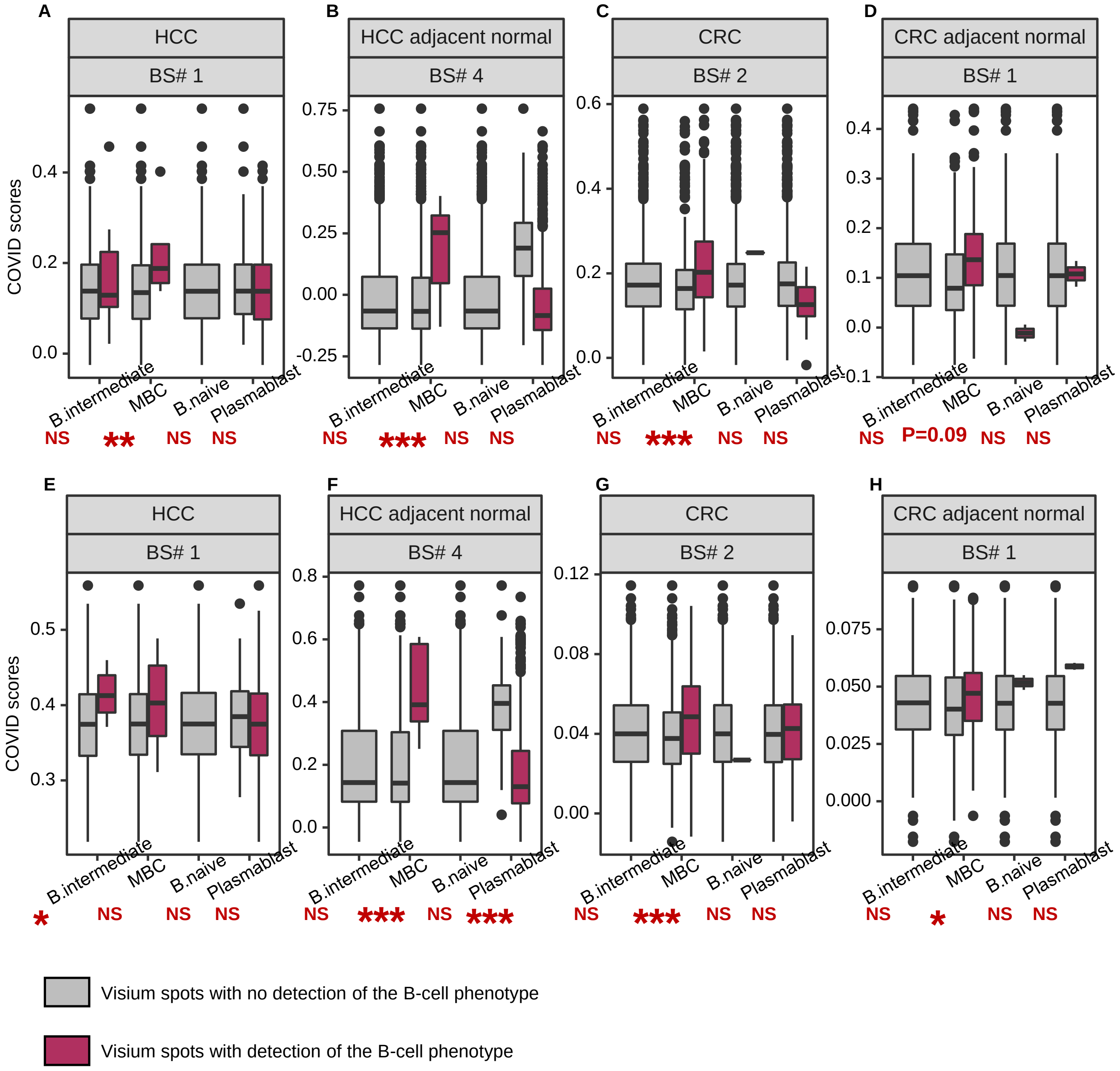

Supplementary Figure 16

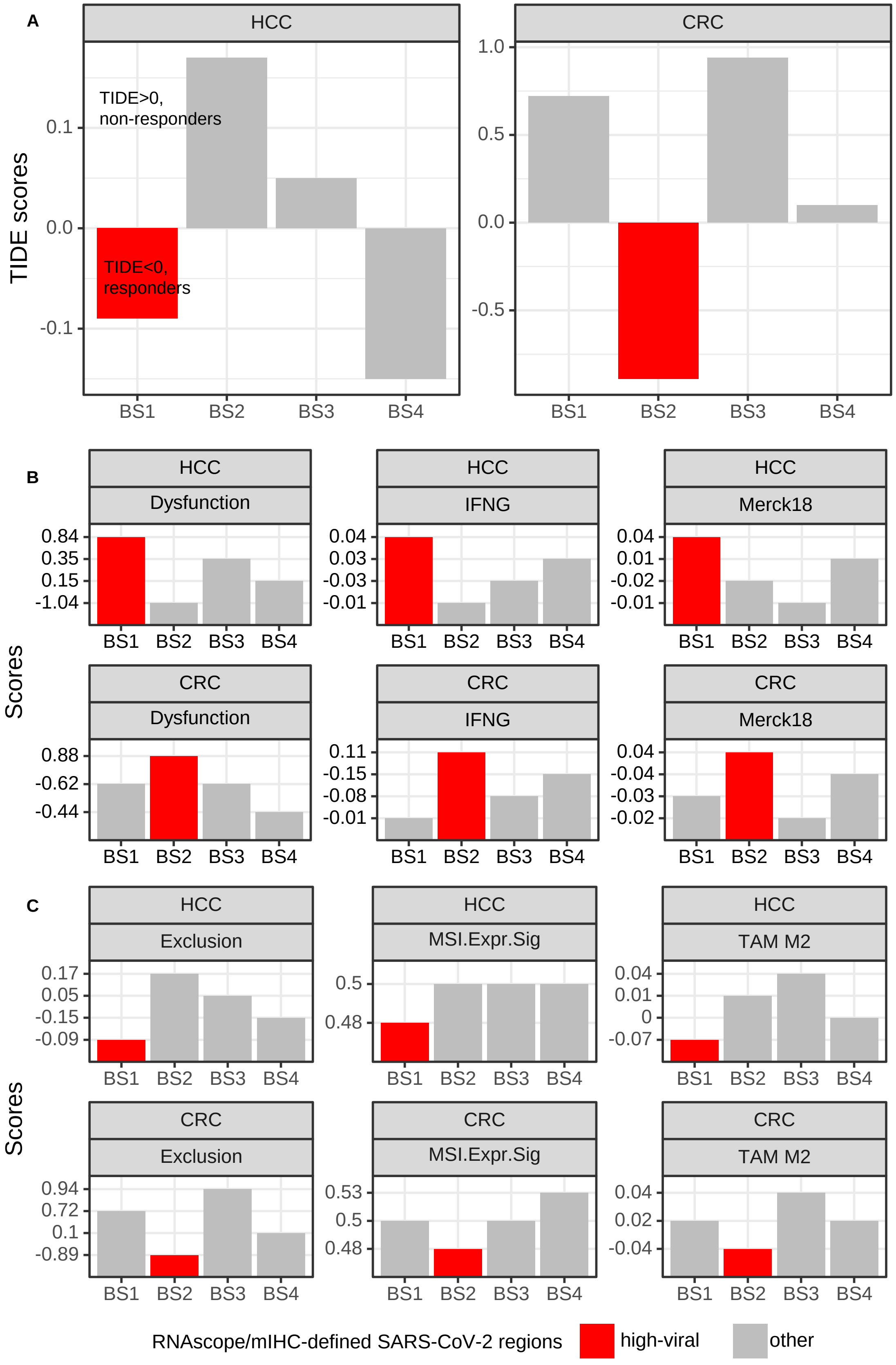

Supplementary Figure 17

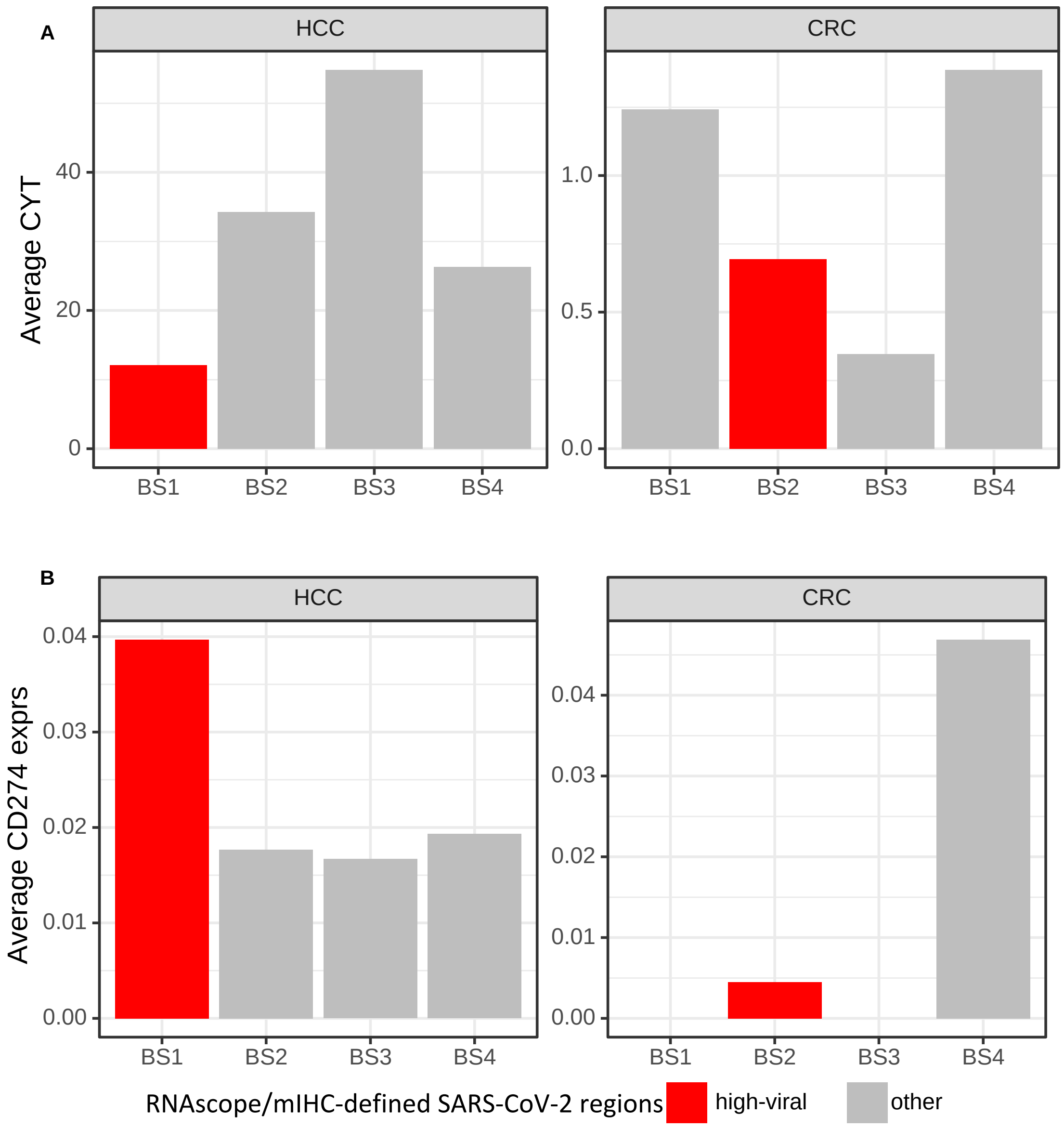

Supplementary Figure 18

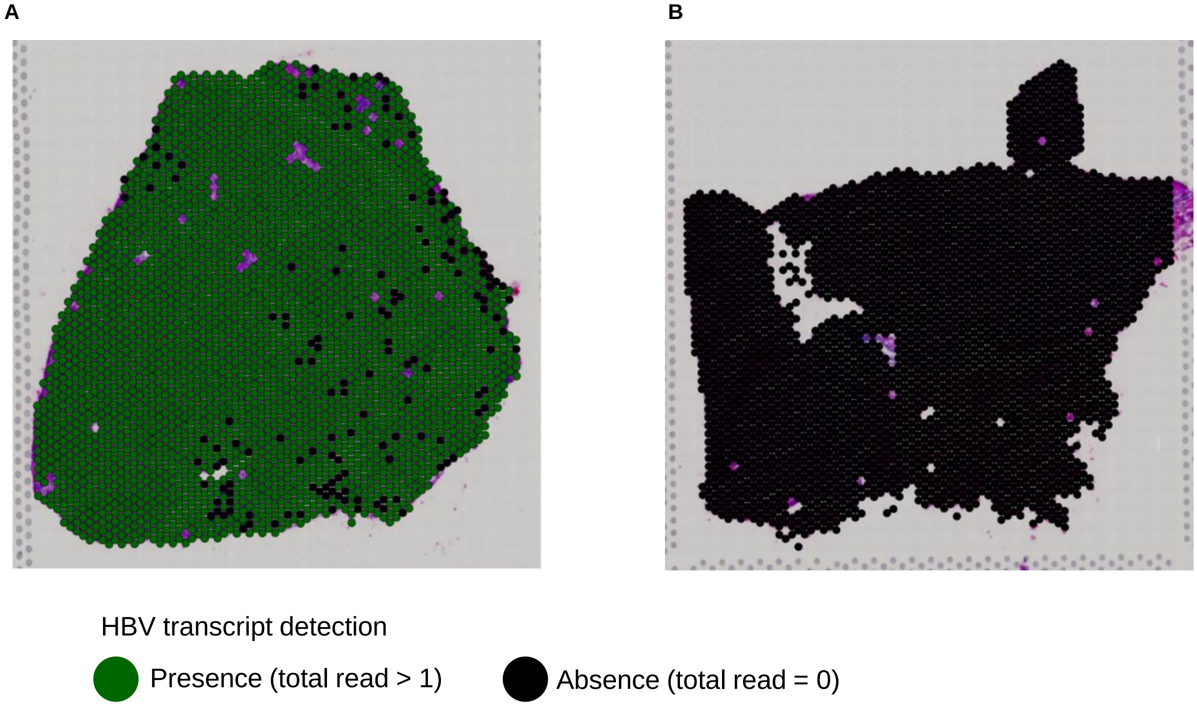

Supplementary Figure 19
